# Treatment of Inguinal Lymph Node Metastases in Patients with Rectal Adenocarcinoma

**DOI:** 10.1245/s10434-019-07191-4

**Published:** 2019-02-06

**Authors:** J. A. W. Hagemans, J. Rothbarth, G. H. W. van Bogerijen, E. van Meerten, J. J. M. E. Nuyttens, C. Verhoef, J. W. A. Burger

**Affiliations:** 1 Department of Surgical Oncology, Erasmus MC Cancer Institute, Rotterdam, The Netherlands; 2 Department of Radiation Oncology, Erasmus MC Cancer Institute, Rotterdam, The Netherlands; 3 Department of Medical Oncology, Erasmus MC Cancer Institute, Rotterdam, The Netherlands; 4 Department of Surgery, Catharina Hospital Eindhoven, Eindhoven, The Netherlands

## Abstract

**Background:**

Inguinal lymph node metastases (ILNM) from rectal adenocarcinoma are rare and staged as systemic disease. This study aimed to provide insight into the treatment and prognosis of ILNM from rectal adenocarcinoma.

**Methods:**

All patients with a diagnosis of synchronous or metachronous ILNM from rectal adenocarcinoma between January 2005 and March 2017 were retrospectively reviewed.

**Results:**

The study identified 27 patients with ILNM (15 with synchronous and 12 with metachronous disease). After discussion by a multidisciplinary tumor board, 19 patients were treated with curative intent, 17 of whom underwent inguinal lymph node dissection. Of the 17 patients, 12 had locally advanced rectal cancer (LARC) with isolated ILNM, 3 had LARC and metastases elsewhere, and 2 had locally recurrent rectal cancer (LRRC). The median overall survival (OS) for all the patients treated with curative intent was 27 months [95% confidence interval (CI) 11.6–42.4 months], with a 5-year OS rate of 34%. The median OS for the patients with LARC and isolated ILNM (*n *= 12) was 74 months (95% CI 18.0–130.0 months), with a 5-year OS rate of 52%. All the patients with metastases elsewhere (*n* = 3) or LRRC (*n* = 2) experienced recurrent systemic disease. Eight patients were treated with palliative intent. The median OS for this group was 13 months (95% CI 1.9–24.1 months), with a 3-year OS rate of 0%.

**Conclusion:**

Clinicians should not consider ILNM as an incurable systemic disease. Patients with primary rectal cancer and solitary ILNM who were eligible for curative surgical treatment had a 5-year survival rate of 52%. The prognosis for patients with additional systemic metastases or LRRC is worse, and the benefit of surgery is unclear.

Locally advanced rectal cancer is associated with pelvic lymph node metastases inside and sometimes outside the mesorectum. Besides these locoregional lymph node metastases, inguinal lymph node metastases (ILNM) may occur, particularly in lower rectal cancer, due to the lymphatic drainage by inguinal lymph nodes.^1^ These ILNMs are relatively rare, and the number of patients described in the literature is low.^2^^–^7

The American Joint Committee on Cancer (AJCC) Cancer Staging Manual considers ILNM from rectal cancer as a systemic disease.^8^ Whether ILNM should be treated with palliative or curative intent is unclear.^9^^–^11 Obviously, patients with ILNM have a worse prognosis than patients without ILNM, but even patients with lung or liver metastases are not always restrained from curative treatment.^12^ The evidence in the literature whether patients with ILNM from rectal adenocarcinoma can possibly be cured is scarce, and few studies have described treatment for ILNM of rectal cancer.^2^^,^^4^^–^6

At our hospital, ILNM has been treated by inguinal lymph node dissection (ILND), with and without neoadjuvant chemotherapy, in case there were no other metastases or when limited metastases were present elsewhere. This report presents the results for patients treated with both curative and palliative intent for ILNM from rectal cancer.

## Methods

All consecutive patients with ILNM from rectal adenocarcinoma treated at the Erasmus MC Cancer Institute, a tertiary referral center in the Netherlands, between January 2005 and March 2017, were retrospectively identified by a search in the local pathology and rectal cancer database. All patients with synchronous or metachronous ILNM were included in the study. Patients with deep/iliac groin nodes were not included.

Patient characteristics, collected from medical records, included tumor characteristics, treatment, surgical variables, short- and long-term outcomes, and postoperative mortality and morbidity. All the patients were followed up at our institution, and the last update of follow-up evaluation was 24 April 2018. Approval for this study was granted by the local medical ethics committee (Registration No. MEC-2017-448).

Synchronous ILNMs were defined as all ILNMs diagnosed before surgery for the primary rectal tumor. Metachronous ILNMs were defined as all ILNMs diagnosed after surgery. All the patients with suspicious ILNMs during physical examination or on imaging [computed tomography (CT) of the abdomen or magnetic resonance imaging (MRI) of the pelvis] underwent lymph node biopsy.

All the patients were screened for disseminated disease by CT of the thorax and abdomen. All the patients were discussed by a multidisciplinary tumor board before treatment and were assessed for eligibility to receive treatment with curative or palliative intent.

Neoadjuvant (chemo)radiotherapy usually comprised a cumulative dose of 50 Gy for primary rectal cancer and a cumulative dose of 30 Gy for LRRC in fractions of 1.8–2.0 Gy, both with concomitant oral chemotherapy (capecitabine 825–1000 mg/m^2^ for 5–7 days a week). The target volume (95% of the radiation dose) mainly was the rectum, but inguinal nodes often received a substantial percentage (~ 30–50%) of the radiation dose. Neoadjuvant induction chemotherapy for ILNM was incidentally given.

For the patients with synchronous ILNM who underwent surgical treatment, an inguinal lymph node dissection (ILND) was performed either simultaneously with surgery for the rectal tumor or upfront before the start of neoadjuvant treatment for the rectal tumor. In case of metachronous metastases, an ILND was performed, in some cases simultaneously with surgical removal of a local recurrence. Notably, only superficial groin dissections were performed.

### Statistical analysis

Data are reported as median [interquartile range (IQR) or 95% confidence interval] or mean ± standard deviation as appropriate. Categorical data are reported as count (%). The Kaplan–Meier method was used for survival analysis, and a log rank test was performed for comparison. The median follow-up period was calculated with the reversed Kaplan–Meier method. Overall survival was calculated from the day ILNM was diagnosed until death or the date of the last follow-up visit. Statistical analysis was performed using IBM SPSS Statistics version 24.0.0 for Windows (IBM Corp, Armonk, New York, USA).

## Results

**Fig. 1:**
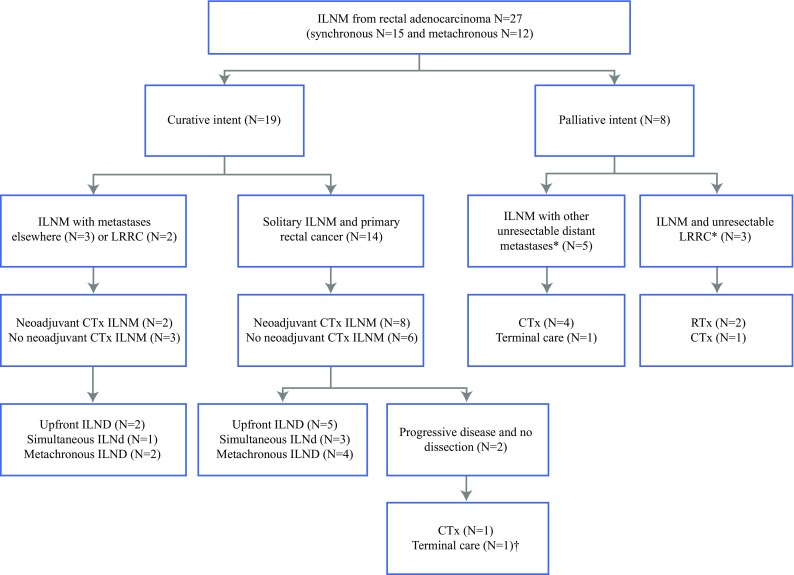
Flowchart included patients. *ILNM* inguinal lymph node metastases, *ILND* inguinal lymph node dissection, *LRRC* locally recurrent rectal cancer, *CTx* chemotherapy, *RTx* radiotherapy, Upfront, upfront dissection before resection of rectal tumour; Simultaneous, simultaneous resection with rectal tumour, *Metachronous*, resection during follow up rectal tumour.*Reason palliative treatment; †Died of respiratory failure before treatment

A flowchart of study patients is shown in Fig. 1. Patient and primary tumor characteristics are listed in Table 1. The characteristics of ILNM and follow-up evaluation are shown in Table 2. The study identified 27 patients with ILNM from rectal adenocarcinoma. The majority of the ILNMs were from low rectal cancer (82%). The median age at diagnosis of ILNM was 63 years (IQR 44–69 years). The median interval between diagnosis of the primary tumor and diagnosis of ILNM was 6 months (IQR 1–30 months). All the patients were discussed by a multidisciplinary tumor board, after which 19 patients were treated with curative intent and 8 patients with palliative intent.

**Table 1:** Patient and primary tumor characteristics

	Total	Curative intent	Palliative intent
	*n* = 27 *n* (%)	*n* = 19 *n* (%)	*n* = 8 *n* (%)
Gender			
Male	12 (44)	7 (73)	3 (38)
Female	15 (56)	12 (63)	5 (63)
Age at ILNM diagnosis			
Median (IQR)	63 (44–69)	60 (40–69)	64 (57–67)
ASA			
ASA 1–2	25 (93)	18 (95)	7 (78)
ASA > 2	2 (7)	1 (5)	1 (13)
Rectal tumor at ILNM diagnosis			
Primary	21 (78)	17 (90)	4 (50)
LRRC	6 (22)	2 (11)	4 (50)
Distance from anal verge (cm)			
Median (IQR)	2 (1–3)	1 (0–7)	2 (1–3)
Location of rectal tumor			
Low rectal (< 5 cm)	22 (82)	14 (74)	8 (100)
Mid rectal (5–10 cm)	3 (11)	3 (16)	0 (0)
High rectal (> 10 cm)	2 (7)	2 (11)	0 (0)
Neoadjuvant therapy for rectal tumor			
CTxRTx	18 (67)	14 (74)	4 (50)
RTx	4 (15)	3 (11)	1 (13)
CTx	0 (0.0)	0 (0)	0 (0)
No neoadjuvant therapy	5 (19)	2 (11)	3 (38)
Surgical procedure for primary tumor			
No resection^a^	2 (5)	2 (11)	0 (0)
LAR	7 (26)	4 (21)	3 (38)
APR	9 (33)	5 (26)	4 (50)
APR with HIPEC	1 (4)	1 (5)	0 (0)
Posterior pelvic exenteration	4 (15)	3 (16)	1 (13)
Total pelvic exenteration	4 (15)	4 (21)	0 (0)
Tumor stage of primary tumor			
No resection	2 (8)	2 (11)	0 (0)
T2	3 (11)	2 (11)	1 (13)
T3	11 (41)	7 (37)	4 (50)
T4	11 (41)	8 (42)	3 (38)
Nodal stage of primary tumor			
No resection	2 (7)	2 (11)	0 (0)
N0	10 (37)	5 (26)	5 (63)
N1	8 (30)	6 (32)	2 (25)
N2	7 (26)	6 (32)	1 (13)

Numbers do not add up due to rounding

*ILNM* inguinal lymph node metastases, *IQR* interquartile range, *ASA* American Society of Anesthesiology, *LRRC* locally recurrent rectal cancer, *CTxRTx* chemoradiotherapy, *RTx* radiotherapy, *CTx* chemotherapy, *LAR* low anterior resection, *APR* abdominoperineal resection, *HIPEC* hyperthermic intraperitoneal chemotherapy

^a^No resection due to progressive disease

**Table 2:** Inguinal lymph node metastases and histopathologic characteristics and follow-up evaluation

	Total	Curative intent	Palliative intent
	*n* = 27 *n* (%)	*n* = 19 *n* (%)	*n* = 8 *n* (%)
Time from Dx of rectal cancer until ILNM			
Median months (IQR)	6 (1–30)	4 (0–4)	24 (4–56)
Onset of ILNM			
Synchronous	15 (56)	13 (68)	2 (25)
Metachronous	12 (44)	6 (32)	6 (75)
Location of ILNM			
Unilateral	19 (70)	14 (74)	5 (63)
Bilateral	8 (30)	5 (26)	3 (38)
Solitary ILNM			
No	8 (30)	3 (16)	5 (63)
Yes	19 (70)	16 (84)	3 (38)
Distant metastases elsewhere			
Liver	1 (4)	1 (5)	0 ()
Lung	1 (4)	0 ()	1 (13)
Peritoneal	2 (7)	1 (5)	1 (13)
Iliac lymph nodes and paraaortic	1 (4)	0 (0)	1 (13)
Lung and spinal bone	1 (4)	0 (0)	0 ()
Liver and iliac lymph nodes	2 (7)	1 (5)	1 (13)
Lung and iliac lymph nodes	2 (7)	0 (0)	1 (13)
Neoadjuvant CTx for ILNM			
No	17 (63)	9 (47)	N/A
Yes	10 (27)	10 (53)	N/A
ILND			
No dissection	10 (37)	2 (11)	8 (100)
Upfront	7 (26)	7 (37)	0 (0)
Simultaneous with rectal tumor	4 (15)	4 (21)	0 (0)
Metachronous during FU of rectal cancer	6 (22)	6 (37)	0 (0)
Histopathology of inguinal lymph nodes specimen^a^			
Positive lymph nodes			
No	NA	4 (24)	NA
Yes	NA	13 (76)	NA
Total no. of harvested nodes			
Median (range)	NA	12 (3–16)	NA
Total no. of positive nodes			
Median (range)	NA	1 (0–11)	NA
Follow-up after surgical treatment			
Disease status at last follow-up			
No evidence of disease	NA	5 (29)	NA
Distant metastases	NA	7 (41)	NA
Local recurrence of rectal cancer and	NA	7 (41)	NA
Distant metastases			
Inguinal lymph node recurrence^b^	NA	2 (12)	NA

Numbers do not add up due to rounding

*Dx* diagnosis, *ILNM* inguinal lymph node metastases, *IQR* interquartile range, *CTx* chemotherapy, *ILND* inguinal lymph node dissection, *FU* follow-up, *NA* not applicable

^a^17 patients and 22 dissection specimens, due to five bilateral ILN

^b^Nodal recurrence in dissected site

### Curative intent

For 10 of the 19 patients treated with curative intent, neoadjuvant chemotherapy for ILNM was administered, and all the patients received (chemo)radiotherapy for the rectal tumor. For two patients, the target volume included the ILNM. In all the remaining patients, the inguinal nodes received a lower percentage (30–50%) of the total radiation dose.

Two patients with primary rectal cancer had progression of disease during neoadjuvant chemotherapy and were then treated palliatively, as depicted in Fig. 1. Subsequently, ILND was performed for 17 patients. Of these 17 patients,^12^ had primary locally advanced rectal cancer and solitary ILNM, 3 had metastases elsewhere (liver, *n* = 2; peritoneal, *n* = 1), and 2 patients had locally recurrent rectal cancer.

### Palliative intent

Eight patients were treated with palliative intent for disseminated disease or unresectable LRRC using chemotherapy, radiotherapy, or terminal care, as displayed in Fig. 1. Five of these patients had received neoadjuvant radiotherapy for the rectal tumor, and the ILNMs were outside the target volume but still received a lower percentage (30–50%) of the total radiation dose. Two patients had received palliative radiotherapy with ILNM receiving the target volume, a dose of 32 and 45 Gy, respectively.

### Mortality and morbidity

#### Curative intent

None of the patients died within 30 days of surgery, and 6 (35%) of the 17 patients experienced postoperative complications. Four patients experienced inguinal seroma despite the standard use of postoperative suction drainage, which required percutaneous drainage in all cases. Two patients used antibiotics to treat superficial wound infections. Two patients experienced lymphedema during the follow-up period and required elastic compression garments.

Of all the patients with inguinal complications, one patient had received neoadjuvant radiotherapy specifically on the inguinal nodes. In all the remaining patients, the inguinal nodes were outside the target area but still partly inside the radiotherapy field.

#### Palliative intent

Half of the patients who received palliative treatment had ILNM-related morbidity. Four patients experienced severe pain requiring intravenous pain medication, and three of these patients also had lymphedema. One patient experienced lymphedema without complaints. Four patients with lymphedema had received radiotherapy for the rectal tumor, with inguinal nodes partly in the radiation field. Two of these patients also had received a high-dose palliative radiotherapy specifically on the inguinal nodes, but already had experienced lymphedema before palliative radiotherapy.

### Histopathologic results after ILND

Histopathologic evaluation was performed for 22 dissection specimens from 17 patients. The median number of lymph nodes found was 12 (range 3–26), and the median number of positive lymph nodes was 1 (range 0–11).

In four patients treated with curative intent, no positive lymph nodes were found. Three of these four patients had received neoadjuvant chemotherapy and were considered complete responders. In one patient without neoadjuvant therapy, three negative nodes were recovered, but four tumor deposits in the specimen were found, and this patient experienced local and distant recurrence during the follow-up period. In the remainng 13 patients, positive lymph nodes were found. Of these 13 patients, 5 had received neoadjuvant chemotherapy for ILNM.

### Follow-up evaluation, recurrence, and survival

The median follow-up period for the survivors in the total cohort of 27 patients was 79 months (95% CI 46.9–111.1 months), during which 20 patients died. The median overall survival for the total cohort was 19 months (95% CI 5.8–32.2 months). There was no significant difference in survival between synchronous or metachronous ILNM (*p* = 0.86) and bilateral or unilateral ILNM (*p* = 0.05).

#### Curative intent

Of 19 patients treated with curative intent, 2 had progressive disease under neoadjuvant therapy and experienced distant metastases, whereas the primary rectal tumor and the ILNM remained in situ. At the last follow-up visit, 5 of the 17 patients who underwent ILND had no evidence of disease. Of these 17 patients, 2 experienced a local ILNM recurrence, accompanied by local recurrence of the rectal tumor and systemic metastases. Another five patients experienced local recurrence of the rectal tumor with distant metastases, and five patients experienced distant metastases alone.

At the last follow-up visit, seven patients were alive, and all these patients had undergone ILND. Three patients were alive with local rectal tumor recurrence and distant metastases, including one patient with ILNM recurrence. Four patients were alive with no evidence of disease, and one patient had died with no evidence of disease.

**Fig. 2:**
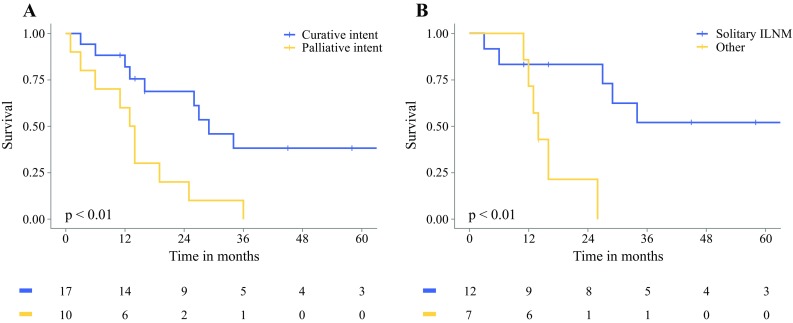
Overall survival. **a** Curative intent versus palliative intent; **b** Patients with curative intent: Solitary ILNM with primary LARC vs Other (ILNM with other metastases *N* = 3 or LRRC *N* = 2 or progressive disease under chemotherapy *N* = 2)

Survival curves are shown in Fig. 2. The median overall survival for all 19 patients treated with curative intent after diagnosis was 27 months (95% CI 11.6–42.4 months). The 1- and 5-year estimated overall survival rates were respectively 79% and 34%.

For 12 patients with solitary ILNM from primary rectal cancer without systemic metastases who underwent curative ILND, the median overall survival period was 74 months (95% CI 18.0–130.0 months) with 1- and 5-year estimated overall survival rates of 83% and 52%, respectively.

Three patients underwent ILND with resection of the primary rectal tumor and resection of metastases elsewhere (liver, *n* = 2; peritoneal, *n* = 1). Two of these patients died of systemic disease at 13 and 26 months of follow-up evaluation, respectively, and one patient, who underwent ILND and surgery for primary rectal cancer with liver metastases only, at this writing is still alive after 14 months of follow-up evaluation with locally recurrent rectal cancer and recurrent liver metastases. The two patients who underwent ILND with simultaneous resection of locally recurrent rectal cancer died of systemic disease with respectively 12 and 13 months of follow-up evaluation.

#### Palliative treatment intent

At the last follow-up visit, all eight patients treated with palliative intent had died of the disease. The median overall survival was 13 months (95% CI 1.9–24.1 months), with 1- and 3-year estimated overall survival rates of 63% and 0%, respectively.

## Discussion

In this study, a 5-year survival rate of 52% was achieved after surgical treatment of patients with primary rectal cancer. An isolated ILNM prognosis for patients with additional systemic metastases is worse, and the benefit of surgery is unclear. Patients treated with curative surgery mostly experienced lymphedema, and palliatively treated patients mostly had severe pain.

In 1990, Graham and Hohn^7^ were among the first to describe management of ILNM. Their study identified 40 patients with ILNM from rectal cancer divided into three groups: (1) unresectable primary tumors, (2) recurrent disease after abdominoperineal resection with palliative treatment, and (3) solitary ILNM treated with ILND. None of the patients survived 5 years, but the median survival was highest in the solitary ILNM group (inguinal metastases only), with two patients having no evidence of disease at the last follow-up visit (one patient died of myocardial infarction, and one patient was alive with 15 months of follow-up evalution). These authors concluded that only in case of solitary ILNM, the situation for eight patients in their study, a resection may be warranted.

Tocchi et al.^4^ reported a mean, not median, survival of 14.8 months for 21 patients with ILNM from rectal cancer, and none of the patients reached 5-year survival. Their study included five patients with ILNM only and supported the suggestion that ILND can be beneficial, although not curative, because a prolonged survival was achieved for these patients. They concluded that ILNM is frequently associated with distant metastases (in 16 of 21 patients of their series), and in these cases, systemic therapy is the treatment of choice.

Luna-Pérez et al.^6^ described a 5-year survival for 0% of 32 patients with ILNM from rectal adenocarcinoma, 27 of whom also had systemic metastases. They concluded that surgery for isolated ILNM may prolong survival, but that ILNM should be considered as systemic disease and treated palliatively as indicated.

More recent studies by Bardia et al.^2^ and Adachi et al.^5^ retrospectively reviewed small groups of patients with ILNM and concluded that patients with isolated ILNM are a different subset of patients. Bardia et al.^2^ studied six patients with solitary ILNM, and the mean survival period for these patients was 40 months. Adachi et al.^5^ studied 10 patients with ILNM, 8 of whom had solitary ILNM and underwent ILND. They reported a 5-year overall survival rate of 75% for these patients. Adachi et al.^5^ also reported a better prognosis for patients with metachronous metastases, but our study did not find any difference in survival between metachronous and synchronous metastases. This may be explained by the definitions Adachi et al.^5^ used for synchronous (up to 1 year after diagnosis of the primary rectal cancer) and metachronous metastases (> 1 year after diagnosis of primary rectal cancer) or by the small number of patients in both studies.

The current study presents the largest group of patients with ILNM caused by rectal cancer who were treated with curative intent since the study by Luna-Pérez et al.^6^ in 1999. However, the majority of the patients in the latter study had distant metastases as well and may not be considered candidates for curative treatment. The results of previous studies presenting smaller groups of patients are confirmed: ILNM caused by rectal cancer should not necessarily be considered an incurable disease, especially in case of primary rectal cancer and the absence of other systemic metastases. In our study, a median OS of 74 months with 1- and 5-year estimated overall survival rates of 83% and 52%, respectively, was reached for this group.

In line with all other previously published studies, our study showed a poor prognosis for the patients with ILNM and distant metastases to other sites.^2^^,^^4^^–^7 These results suggest that these patients should be treated with palliative intent.

The current study included three patients who underwent resection of ILNM combined with resection of additional metastases. Only one patient, who underwent ILND and liver metastases resection, at this writing is still alive at 14 months follow-up evaluation, with systemic recurrence. In addition, both patients with locally recurrent rectal cancer who underwent resection of the rectal tumor with ILND died within 13 months. Due to small numbers, no conclusions can be drawn, and it is unclear whether surgery was at all beneficial for these patients. Currently, in the Netherlands, the ORCHESTRA trial is being performed to assess the beneficial effects of added local treatment with systemic treatment in case of systemic disease and possibly will provide evidence in the future.^13^

The mortality and morbidity associated with ILND have been described for ILNM caused by melanoma and anal cancer, but few studies have described morbidity after ILND for rectal cancer.^14^^–^16 The mortality is low, but the morbidity associated with this procedure is high. Short-term wound complications such as dehiscence, infection, and seroma are reported to reach 60%, and lymphedema can occur.^14^^–^16

In our study, 6 (35%) of 17 patients experienced postoperative complications. All the patients with inguinal complications had received (partial) prior irradiation on inguinal nodes. The numbers were small in the current study, but the negative impact of radiation therapy is well known. Radiation therapy impairs wound healing and can increase the incidence of lymphedema.^17^ Recent series indicate that routine inguinal lymph node radiation is not necessary.^17^^,^^18^ The optimal balance between inguinal radiotherapy and the extent of surgery is unclear, but the morbidity of the combined procedure should not be underestimated.

In the current study, only ILND (i.e., superficial groin dissections) were performed and no deep groin dissection. In 12 of the 17 patients who underwent ILND, distant metastases occurred outside the pelvic region, including four patients with simultaneous iliac node recurrence. This could imply that a formal deep groin dissection in all these patients for a superficial ILNM would have been overtreatment with considerable morbidity.

Our study was limited by its small numbers, referral bias from other centers, and its retrospective nature. Inguinal lymph node metastases from rectal adenocarcinoma are relatively rare, and most previous studies contain small and heterogeneous groups of patients collected before the era of total mesorectal excision (TME) surgery and before neoadjuvant therapy was widely accepted. Although the current study presents a small cohort, it provides proof that solitary ILNM from rectal adenocarcinoma does not equal incurable disease. This is supported by previous reports.

## Conclusion

Surgical treatment of ILNM from rectal adenocarcinoma may result in prolonged survival and possibly a cure. Inguinal lymph node metastases should not be considered as an incurable disease, especially in patients with primary rectal cancer and solitary ILNM. The prognosis for patients with ILNM and distant metastases elsewhere or recurrent rectal cancer is worse, and the value of surgery is unclear.
